# Geographical and temporal variation in environmental conditions affects nestling growth but not immune function in a year-round breeding equatorial lark

**DOI:** 10.1186/s12983-017-0213-1

**Published:** 2017-05-26

**Authors:** Henry K. Ndithia, Samuel N. Bakari, Kevin D. Matson, Muchane Muchai, B. Irene Tieleman

**Affiliations:** 1 Ornithology Section, Department of Zoology, National Museums of Kenya, P.O. Box 40658 –00100 GPO, Nairobi, Kenya; 2 Groningen Institute for Evolutionary Life Sciences, University of Groningen, P.O. Box 11103, 9700 CC Groningen, The Netherlands; 3 Resource Ecology Group, Department of Environmental Sciences, Wageningen University, Droevendaalsesteeg 3a, 6708 PB Wageningen, The Netherlands; 4 Department of Clinical Studies (Wildlife and Conservation), College of Agriculture and Veterinary Sciences, University of Nairobi, Box 30197–00100, Nairobi, Kenya

**Keywords:** Breeding, Environment, Growth, Lark, Immunity, Weather

## Abstract

**Background:**

Variation in growth and immune function within and among populations is often associated with specific environmental conditions. We compared growth and immune function in nestlings of year-round breeding equatorial Red**-**capped Lark *Calandrella cinerea* from South Kinangop, North Kinangop and Kedong (Kenya), three locations that are geographically close but climatically distinct. In addition, we studied growth and immune function of lark nestlings as a function of year**-**round variation in breeding intensity and rain within one location. We monitored mass, wing, and tarsus at hatching (day 1) and at 4, 7, and 10 days post**-**hatch, and we quantified four indices of immune function (haptoglobin, agglutination, lysis and nitric oxide) using blood samples collected on day 10.

**Results:**

Nestling body mass and size at hatching, which presumably reflect the resources that females allocated to their eggs, were lowest in the most arid location, Kedong. Contrary to our predictions, nestlings in Kedong grew faster than nestlings in the two other cooler and wetter locations of South and North Kinangop. During periods of peak reproduction within Kedong, nestlings were heavier at hatching, but they did not grow faster over the first 10 days post**-**hatch. In contrast, rainfall, which did not relate to timing of breeding, had no effect on hatching mass, but more rain did coincide with faster growth post**-**hatch. Finally, we found no significant differences in nestling immune function, neither among locations nor with the year**-**round variation within Kedong.

**Conclusion:**

Based on these results, we hypothesize that female body condition determines nestling mass and size at hatching, but other independent environmental conditions subsequently shape nestling growth. Overall, our results suggest that environmental conditions related to food availability for nestlings are relatively unimportant to the timing of breeding in equatorial regions, while these same conditions do have consequences for nestling size and growth.

## Background

Patterns of growth and development, and ontogeny of immune function vary widely among avian species and populations, variation that is hypothesized to reflect adaptation to specific environmental conditions [1, 2]. Growth rates are associated with pace**-**of**-**life, with faster growth rates associated with species and populations that live at high latitudes [3–7], at high altitudes [8, 9], and in less arid environments [10, 11]. For a given pace**-**of**-**life, i.e. within populations and within seasons, early**-**hatched broods have been shown to grow faster than late**-**hatched broods due to changes in food abundance and quality of diet [12–14]. Like growth rates, immune function has also been hypothesized to vary with pace**-**of**-**life in birds, with reduced investment in the immune system associated with a faster pace**-**of**-**life [5, 15, 16]. However, several studies show that environmental conditions can be more important determinants of immune function than pace**-**of**-**life [17–21]. In addition, within a given pace**-**of**-**life, immune function is not fixed, but changes seasonally in adult birds [20, 22–24] and nestlings [25–27].

Within equatorial regions, which on the global scale are associated with comparatively slow avian growth rates [3], spatial and temporal variation in climatic conditions still exist. This spatio-temporal variation in climatic condition provides a strong opportunity to understand variation in life-history strategies among tropical locations and species. For example, orography and altitudinal differences can lead to large variation in rainfall and temperature over small geographic distances, and rainfall patterns are often unpredictable within regions [28–30]. This within**-**region variation raises questions about how nestling growth rates have evolved in response to different tropical climates, questions that have generally not been investigated. In general, cool and wet locations are thought to provide more food, promoting faster nestling growth; more arid locations are thought to be food deficient, favoring slower growth [3, 10]. In contrast however, investment in growth rate for nestlings in cool and wet locations is expected to compete with requirements for thermoregulation, possibly reducing growth rate in such locations compared to drier ones [31]. Studies including costs of thermoregulation could identify the relative importance of these factors. Differences in environmental conditions within and between years in a location with fluctuating and inconsistent patterns of rainfall and food availability are likely to promote variation in nestling growth rates [4]. Likewise, nestlings raised in food abundant wet seasons grow faster than those raised in food deficient dry seasons [32], the latter of which result in hatching asynchrony commonly recorded among tropical artricial birds [3, 33–35].

Immune defences of organisms living in a particular environment are expected to match pathogen pressure experienced in that environment [36]. Although high parasite pressures associated with tropical regions might also result in tropical birds having relatively robust immune systems compared to their temperate counterparts, environmental variation within equatorial regions should select for intra**-**tropical variation in immune function in adult and nestling birds. Immune function in young birds may be particularly revealing about the parasite pressures exerted by different locations within a region. Since the time for possible parasite exposure and for subsequent acquired immune responses is short, innate immune function and maternally derived antibodies are proposed to be most important for young birds [1, 37–39]. Innate immune function is hypothesized to relate to pace**-**of**-**life [40–42], and maternally derived antibodies reflect maternal exposure to the local parasite pressures [43, 44]. Assuming that immune function is traded off against reproduction [45, 46], one would expect that breeding under favorable environmental conditions allows females to allocate more resources to nestlings and improve their immune systems indirectly (e.g., via more diverse diet) or directly (e.g., through depositing higher concentrations of maternal antibodies and defenses).

In order to better understand the role of intra**-**tropical variation on patterns of avian growth and development, and on immune function, we exploited the spatial and temporal variation in climate found in equatorial Kenya. This is an understudied component of the pace-of-life debate and one to which this study can strongly contribute. Here, locations that are in close proximity to one another have distinct rainfall and temperature patterns, and within locations, seasonal patterns of rainfall can be unpredictable [30]. Despite these differences, our study species, the Red**-**capped Lark *Calandrella cinerea*, occurs across locations. This provides the opportunity for intraspecific comparisons of environmentally**-**induced variation in nestling growth and immune function. We have previously found that Red**-**capped Larks breed year**-**round, particularly in Kedong, one of our study locations, and that nesting activities fluctuate throughout the year without direct associations with rainfall, temperature or invertebrate abundance [30]. Assuming that a high nesting intensity indicates a favorable set of environmental conditions, this system allows for the study of nestling growth and immune function in relation to temporal variation in environmental conditions that are favorable for females to breed or to rear nestlings.

Our overall objective was to investigate variation in growth and immune function in Red**-**capped Lark nestlings in relation to intra**-**tropical variation in environmental conditions. Specifically, we compared nestling growth rates and immunological indices among three climatically**-**distinct locations. We predicted that nestlings raised in cooler and wetter locations, with expected generally higher food availability, would display faster growth and higher investments in immune indices. In Kedong, we also examined consequences of hatching at different times of the year on growth rates and on immunological indices. In particular, we compared times of the year with more and less nesting activity and with more and less rain. We expected nestlings hatched during times of the year when more larks bred to grow faster and to have more robust immune defences assuming that a high nesting intensity indicates a favorable set of environmental conditions for breeding or rearing nestlings. Because we previously found timing of breeding to be unrelated to rain and food availability [30], we predicted that rainfall would not affect the allocation of resources to nestling growth and immunity.

## Methods

### Study species

The Red**-**capped Lark is a widespread grassland species occurring in large parts of Africa. In Kenya, the species’ distribution ranges from dry and warm lowlands about 1200 m above sea level (a.s.l.) to cool and wet montane grasslands 2600 m a.s.l. [47]. Red**-**capped Larks build open**-**cup nests on the ground often next to a scrub or grass tuft, and lay clutches of two eggs; only two of the 290 nests we found had a clutch of three eggs. They feed on a variety of invertebrates and occasionally on grass seeds. Color ring re**-**sightings suggest that at least part of our study populations is resident to our study locations year round (pers. obs. H.K.N, S.N.B.).

### Study areas and environmental conditions

We conducted our study from January 2011 to March 2014 at three locations in central Kenya: South Kinangop (0^0^42′30″S, 36^0^36′31″E, 2556 m a.s.l.), North Kinangop (0^0^36′55″S, 36^0^30′48″E, 2428 m a.s.l.), and Kedong (0^0^53′37″S, 36^0^23′54″E, 2077 m a.s.l.). In the presented order, the locations experience increasing temperature and decreasing precipitation (Table 1). South and North Kinangop are high altitude montane grasslands that lie along the Aberdare ranges. During and after heavy rains, South Kinangop can experience flooding for some but not all months of the year (normal breeding continues in the absence of flooding) and North Kinangop partial flooding (affecting a few nests), causing damage to nest and death to nestlings [30]. Kedong, a privately owned and extensively grazed ranch in the Rift Valley, consists of large grassland patches that never flood. Direct maximum distances between these locations are 19 km (South Kinangop **-** North Kinangop), 29 km (South Kinangop **-** Kedong) and 34 km (North Kinangop – Kedong). Although we cannot fully exclude the possibility of exchange among locations, we never observed any movements between locations based on the total of 344 color-ringed birds; we observed our birds to be relatively resident. In addition, the three locations are not connected by grassland corridors but in contrast, are separated by natural barriers including escarpment and forest patches that make movement between locations less likely. Within each location, we worked in multiple plots, including Seminis in South Kinangop, Joshua, Mbae and Ndarashaini in North Kinangop and four grassland patches in Kedong. To obtain monthly rainfall in Kedong, we used a weather station (Alecto WS**-**3500, Den Bosch, Netherlands) that measured daily rainfall (mm).

**Table 1:** Annual (*n* = 3 years) and monthly (*n* = 36 months) rainfall (average ± SD, and range), and monthly minimum and maximum temperatures (*n* = 36 months, average ± SD, and range) as measured by our weather stations in South Kinangop, North Kinangop and Kedong, during March 2011 – February 2014 (from Ndithia et al. [30])

Location	Annual rainfall (mm)	Monthly rainfall (mm)		Monthly minimum temperature (°C)		Monthly maximum temperature (°C)	
	Mean ± SD	Mean ± SD	Range	Mean ± SD	Range	Mean ± SD	Range
South Kinangop	939 ± 132.7	78 ± 69.7	0–309	5.5 ± 1.06	3.0–8.2	24.7 ± 2.09	21.2–30.0
North Kinangop	584 ± 62.6	49 ± 35.3	0–155	9.1 ± 2.42	3.0–13.7	25.4 ± 2.27	22.1–30.5
Kedong	419 ± 96.8	35 ± 39.2	0–153	10.5 ± 1.92	6.2–15.7	28.6 ± 2.44	25.3–34.9

### Fieldwork: nest search, nestling growth, and nesting index

Searching year round over the entire study period, we found a total of 290 nests: 74 in South Kinangop, 63 in North Kinangop, and 153 in Kedong (for distributions over time, see [30]. Because of high nest loss through predation, flooding, and other causes, sample sizes of nests with nestlings varied by location and with nestling age (see Table 2 for details). We aimed to find nests at the construction or egg stage, and we monitored nests daily or every other day to determine with certainty hatching dates and nestling order. We made extra effort around hatching date to visit nests to establish hatching order. In cases when we did not distinguish first from second-hatched nestling because both hatched before we could make the distinction, we scored 1.5 for both unknown nestlings. At hatching, we clipped the tip of the claw of the hind toe of the first**-**hatched nestling to distinguish first**-** and second**-**hatched nestlings, which generally hatched a few hours apart. For 19 nests that we found already with nestlings, we estimated age of nestlings based on morphological characteristics, including presence of downy feathers and openness of the eyes.

**Table 2:** Clutch sizes, sample sizes of nests and nestlings for growth measurements, and sample sizes of nests and nestlings for immune function of Red**-**capped Lark *Calandrella cinerea* in South Kinangop, North Kinangop and Kedong, three Kenyan locations with a gradient in climatic conditions, during January 2011 to March 2014. Nestlings normally fledged at day 10 or within one or few days thereafter

	Clutch size		Number of nests for growth (number of nestlings)				Number of nests for immune indices (number of nestlings)
Location	Mean ± sd	Range (number of nests)	Day 1	Day 4	Day 7	Day 10	Day 10
South Kinangop	1.8 ± 0.39	1**–**2 (62)	13^a^ (19)	11 (17)	12 (18)	9 (14)	9 (14)
North Kinangop	1.9 ± 0.33	1**–**2 (49)	8 (14)	10 (18)	8 (14)	10 (17)	9 (15)
Kedong	1.9 ± 0.35	1**–**3 (133)	23 (44)	19 (36)	15 (29)	11^b^ (19^b^)	12 (18)

^a^number of nests for mass = 12

^b^number of nests for wing = 10; number of nestlings for wing = 18

We measured body mass, wing length, and tarsus length of nestlings at days 1 (hatching), 4, 7 and 10. We measured body mass using a 50 g Pesola (accuracy, 0.1 g), measured wing length on a flattened and straightened wing using a 150 mm ruler specially designed for measuring birds (accuracy, 0.5 mm) and measured tarsus length from the knee to the base of the last complete scale before the toes diverge using a Vernier calipers (accuracy, 0.1 mm) [48, 49]. Three field assistants and H.N. worked in all three locations and took these measurements on birds. In addition, at the beginning of the project, H.K.N. trained the three assistants to harmonize the measuring skills and avoid observer bias. Nestlings normally fledged between day 10 and 12 of age. On day 7, we trapped both parents using a cage trap at the nest to record morphological parameters.

Red**-**capped Larks breed year**-**round but the number of nests varies from month to month [30]. To quantify the month**-**to**-**month variation in nesting intensity at the population level in Kedong, we calculated a “nest index”: the total number of nests found in a month per 10 h of nest searching effort [30]. From January 2011 to March 2014, our mean monthly search effort in Kedong was 14.1 ± 5.30 days (SD, *n* = 39, range = 7–24 days) or 49.8 ± 35.95 h (SD, *n* = 39, range = 17–193 h).

### Nestling immune function

Using heparinized capillary tubes, we collected blood samples in the field from the brachial wing vein of 47, 10**-**day**-**old nestlings (*n* = 30 nests) in the three locations combined (see Table 2 for breakdown per location). Blood samples were kept on ice and centrifuged at the end of each fieldwork day. The plasma fraction was then frozen for future analyses of haptoglobin, natural antibodies and complement, and nitric oxide.

Haptoglobin, an acute phase protein, increases in concentration in blood in response to acute infection, inflammation, or trauma [50, 51]. We determined the concentration (mg/ml) of haptoglobin (or more specifically, haptoglobin**-**like functional equivalents) using an assay that measures the haem**-**binding capacity of plasma (TP801; Tridelta Development limited, Maynooth, Ireland) following the instructions provided by the manufacturer and with the 5 min incubation step at 30 °C (for details, see [51]). Each of the three assay plates, included an among**-**plate standard which we run in duplicate within each plate [51] (mean within**-**plate coefficient of variation (CV) = 2.4%; mean among**-**plate CV = 2.7%).

Natural antibodies and complement are constitutive components of the innate immune system [52]. We quantified natural antibody**-**induced agglutination and complement**-**induced lysis of rabbit red blood cells (Envigo, Belton, UK) following the protocol of [52]. We scored lysis and agglutination titers from randomized images of assay results. All scoring of lysis and agglutination (HLHA) were done blind to sample and plate identity, and all HLHA samples were scored at least twice by the same person. If the first two scores were <1 titer apart, we used the mean value in statistical analyses. If the difference between the first two scores was >1, we re**-**scored the sample a third time and used the median in analyses. We assigned half scores when samples showed a lysis or agglutination result that was intermediate between two titers. We calculated among**-**plate and within**-**plate variation for agglutination (mean among**-**plate CV = 9.7%; mean within**-**plate CV = 7.7%) and for lysis (mean among**-**plate CV = 18.6%; mean within**-**plate CV = 9.8%).

Nitric oxide is a multifunctional signalling molecule that can provide information about an individual’s condition, and whose functions include the modulation of inflammatory processes and the destruction of parasites, virus**-**infected cells, and tumor cells [53]. We determined nitric oxide production (mmol/ml) through the reduction of nitrate to nitrite following the assay of [53]. We used the Griess reaction assay kit from Promega and recorded absorbance at 542 nm.

### Statistical analyses

We first checked, per location, for differences in mass, wing and tarsus between nestlings whose ages we knew and those whose age we estimated. Over**-**or**-**under estimation of age of nestlings may lead to incorrect data of nestling mass, wing and tarsus. We did not find significant differences between these groups (all t values <2.14, all *P* values >0.07) and therefore pooled them in further analyses. We described growth in mass, wing length and tarsus length using logistic growth curves [11, 54] for each location that we fitted by the R**-**package “car [55].” To compare among locations and ages, we calculated residual values relative to a single overall curve for all locations combined. We fitted this overall curve using average values per age for each location, to account for sample size differences among locations. We expressed residuals as percent deviation from this curve. In further analyses, we used these residuals in linear mixed**-**effects models (“lme” in the R**-**package “nlme”; [56]).

To compare nestling mass, wing length, and tarsus length across locations, we used models with location, age, hatching order and the interaction between location and age as explanatory variables, and with individual nested within nest as random factors. These random factors accounted for the lack of independence between nest mates due to shared genetic background and parental care [57] and repeated measurements on individual nestlings. We used residuals of absolute nestling size (i.e., absolute mass, wing length, or tarsus length) and residuals of relative nestling size (i.e., % of adult mass, wing length, or adult tarsus length) in this comparison. To determine nestling size relative to size at maturity (i.e., % of average adult size), we first calculated sex**-** and population**-**specific mean values of mass, wing length, and tarsus length since we did not know the sex of nestlings. Then we averaged the male and female values to approximate generalized adult values (Appendix). When the interaction between location and age was significant, we ran models per age (day 1, 4, 7, 10) to discover at which age(s) the location effect was significant. We subsequently tested for differences among locations using Tukey post**-**hoc tests.

For the within**-**Kedong analyses of nestling mass, wing length, and tarsus length in relation to nest index and total monthly rainfall, we calculated residuals relative to the logistic curve for Kedong only. Our models contained these residuals as dependent variables; nest index (or rainfall), age, hatching order, and the interaction between nest index and age as explanatory variables; and nest as random factors. We repeated all analyses of mass using instead mass divided by tarsus (an index for body condition); results from the two analyses were similar, so we only report results from the first mass analyses.

For analyses of haptoglobin, we log**-**transformed data, because the residuals of the final model were not normally distributed. For the among location comparison, we first tested and found that sample redness at 450 nm did not affect haptoglobin (F_1, 14_ = 1.13, *P* = 0.31) but sample age did (F_1, 14_ = 6.36, *P* = 0.02) [51]. We then constructed a model that included log haptoglobin as the dependent variable; location, hatching order, sample age, and the interaction between location and hatching order as explanatory variables; and nest as a random factor. For the within Kedong analysis, we established that sample redness (F_1, 5_ = 2.89, *P* = 0.15) and sample age (F_1, 5_ = 2.80, *P* = 0.16) did not significantly affect haptoglobin concentration. Models then included explanatory variables hatching order, and either nest index or monthly rainfall, and the interactions. Again, nest was included as a random factor.

In comparisons of agglutination (log**-**transformed to obtain normality) and nitric oxide across locations, we found no effect of plasma sample age (agglutination F_1, 17_ = 0.06, *P* = 0.81; nitric oxide F_1, 9_ = 0.11, *P* = 0.75). We therefore included location, hatching order, their interaction as explanatory variables, and we included nest as a random factor. For within Kedong analyses, plasma sample age did not affect agglutination (F_1, 6_ = 0.02, *P* = 0.89) or nitric oxide (F_1, 3_ = 0.39, *P* = 0.58) also. We therefore constructed models with nest index or monthly rainfall, hatching order, and the interaction as explanatory variables and nest as a random factor.

Additionally among the three locations, we explored effects of mass and tarsus at day 10 (sample sizes did not allow including growth, and measurement at days 1, 4 and 7) on the three immune measures, because of possible trade-offs between growth and immune measures. We did not find any significant effects of location or mass/tarsus (F_2, 25_ < 1.98, *P* > 0.16); we do not report these results.

For all analyses, we tested and confirmed assumptions about normality and homoscedasticity of variance through graphical and statistical methods. We simplified models using backward elimination by excluding one**-**by**-**one the most insignificant terms (α = 0.05) until we arrived at a final model. We used R statistical software version 3.0.3; [58] in all our analyses.

## Results

### Nestling growth but not immune function varies among three climatically distinct locations

**Fig. 1:**
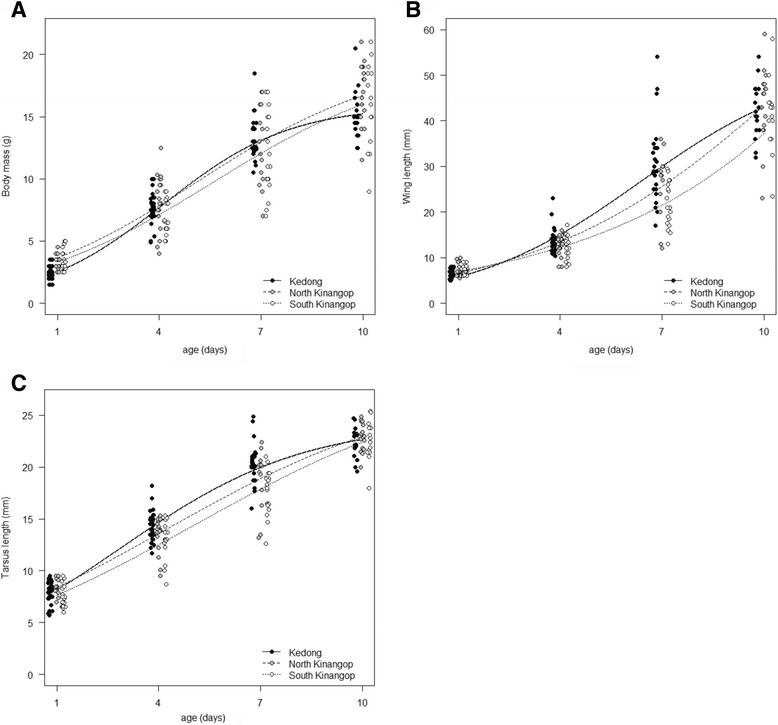
Mass (g, **a**), wing length (mm, **b**) and tarsus length (mm, **c**) of Red**-**capped Lark *Calandrella cinerea* nestlings as a function of age in South Kinangop (*cool and wet*), North Kinangop (warm and wet) and Kedong (*warm and dry*), three Kenyan populations with distinct climates. Data for the three locations are plotted apart to increase visibility rather than that the age at measurement differed between locations

Growth curves for mass and wing differed among locations in a similar fashion with nestlings in Kedong starting at the lowest mass and shortest wing, but having the highest growth constant K for mass and wing, and nestlings in South Kinangop starting at the highest mass and longest wing but having the lowest K for both variables (Fig. 1, Table 3). Comparing residuals among locations for both mass and wing length, we found a significant interaction between location and age, and no significant effect of hatching order (Table 4). Subsequent analyses per age for mass revealed that at hatching (day 1), the location effect was significant (F_2, 40_ = 15.59, *P* < 0.001) and nestling body mass (in g) was 34% higher in South Kinangop than in Kedong (z = 4.98, *P* < 0.001) and 47% higher in North Kinangop than in Kedong (z = 3.85, *P* < 0.001); nestling body masses in South and North Kinangop did not significantly differ from each other (z = 0.46, *P* = 0.89). Among**-**location differences in residual mass on days 4 (F_2, 37_ = 2.02, *P* = 0.15), day 7 (F_2, 32_ = 0.21, *P* = 0.82) and day 10 (F_2, 27_ = 0.91, *P* = 0.42) were not significant.

**Table 3:** Logistic growth curve variables for growing nestlings of Red-capped Larks in South Kinangop, North Kinangop and Kedong, in addition to the overall curve based on the averages of the three locations

	A (S.E.)	t_i_ (S.E.)	K (S.E.)	95% C.I.	K_restricted_ (S.E.)	95% C.I.
Body mass (g)						
South Kinangop	19.5 (2.66)	5.6 (1.00)	0.34 (0.065)	0.21–0.47	0.43 (0.108)	0.21–0.65
North Kinangop	19.7 (2.16)	5.2 (0.82)	0.36 (0.068)	0.21–0.49	0.55 (0.115)	0.29–0.80
Kedong	15.9 (0.38)	4.1 (0.15)	0.54 (0.031)	0.48–0.60	0.57 (0.034)	0.51–0.64
Overall	17.5 (0.59)	4.7 (0.23)	0.43 (0.029)	0.38–0.49		
Wing length (mm)						
South Kinangop	558.9 (3882.94)	23.7 (6.88)	0.19 (0.057)	0.07–0.30	0.21 (0.083)	0.04–0.37
North Kinangop	101.4 (65.63)	11.1 (4.49)	0.26 (0.065)	0.13–0.39	0.37 (0.087)	0.17–0.56
Kedong	53.2 (4.17)	6.4 (0.50)	0.39 (0.037)	0.32–0.46	0.39 (0.074)	0.24–0.54
Overall	72.4 (10.84)	8.8 (1.05)	0.28 (0.060)	0.14–0.41		
Tarsus length (mm)						
South Kinangop	29.0 (3.02)	5.1 (0.97)	0.25 (0.034)	0.18–0.31	0.30 (0.043)	0.21–0.38
North Kinangop	28.0 (2.32)	4.2 (0.75)	0.26 (0.038)	0.18–0.33	0.34 (0.052)	0.22–0.45
Kedong	24.3 (0.60)	2.9 (0.18)	0.37 (0.022)	0.32–0.41	0.36 (0.033)	0.29–0.42
Overall	25.5 (0.71)	3.5 (0.22)	0.30 (0.038)	0.21–0.38		

The logistic function is W(t) = A/(1 + exp. (−K (t - t_i_)), where W(t) is the weight at age t, A is the asymptote of the growth curve, K is the growth rate constant, and t_i_ is the inflexion point or age at maximal growth rate. K_restriced_ is the growth constant when restricting the data set to individuals with repeated measures on days 1, 4, 7 and 10 (Kedong *n* = 10 nestlings, North Kinangop *n* = 3 nestlings, South Kinangop *n* = 10 nestlings). Values in parentheses represent 1 SE. For K, the 95% confidence intervals are given. Hatching day is defined as day 1

**Table 4:** Results of linear mixed**-**effect models examining the relationship of residuals for mass, wing and tarsus lengths as a function of location, age and chick hatching order for nestlings of Red**-**capped Larks *Calandrella cinerea* from South Kinangop, North Kinangop and Kedong

		d.f.	F	P
Mass				
Body mass				
Location*Age		6, 143	10.54	<0.001
Location		2, 57	19.51	<0.001
Age		3, 143	17.97	<0.001
Hatching order		1, 142	0.20	0.66
Wing				
Location*Age		6, 142	9.24	<0.001
Location		2, 58	1.91	0.16
Age		3, 142	11.54	<0.001
Hatching order		1, 141	1.70	0.19
Tarsus				
Location*Age		6, 143	3.62	0.002
Location		2, 58	7.33	0.001
Age		3, 143	2.26	0.08
Hatching order		1, 142	0.11	0.74
NI	1	13	2.31	0.15
Day 8				
NI	1	9	0.33	0.58

Subsequent analyses per age for wing length revealed that wing lengths differed significantly among locations on day 7 (F_2, 32_ = 6.23, *P* = 0.01) but not on days 1 (F_2, 41_ = 1.25, *P* = 0.30), 4 (F_2, 37_ = 0.25, *P* = 0.78) and 10 (F_2, 26_ = 0.17, *P* = 0.85) (Fig. 1). Wing lengths at day 7 were significantly shorter in South Kinangop than in Kedong (z = 3.52, *P* = 0.001), but not significantly different between South and North Kinangop (z = 1.51, *P* = 0.29) or between North Kinangop and Kedong (z = 1.56, *P* = 0.26). Analyses of relative nestling body mass and relative wing length (% of adult mass and wing length, see Appendix for adult masses and wing length) provided qualitatively similar results (not shown).

Tarsus growth curves differed among locations with nestlings in Kedong starting with intermediate tarsus lengths and having the highest K, whereas nestlings in South Kinangop had the shortest tarsi and the lowest K (Fig. 1, Table 3). In the model comparing residuals for tarsus lengths among locations, the interaction between location and age was significant and hatching order was insignificant (Table 4). Analyses per age showed that tarsus lengths differed significantly among locations on days 1 (F_2, 41_ = 3.82, *P* = 0.03) and 7 (F_2, 32_ = 6.58, *P* = 0.004), but not on day 10 (F_2, 27_ = 0.45, *P* = 0.64) (Fig. 1). On day 4, the difference was marginally insignificant for absolute tarsus length (F_2, 37_ = 2.64, *P* = 0.08) and significant for relative tarsus length (F_2, 37_ = 3.89, *P* = 0.03). Results for relative and absolute tarsus length for other ages were qualitatively similar (not shown). Pairwise comparisons among locations showed that tarsus length in South Kinangop was shorter than in Kedong (day 1, z = 2.40, *P* = 0.04; day 7, z = 3.63, *P* < 0.001), tarsus length in South Kinangop was shorter than in North Kinangop only on day 1 (z = 2.40, *P* = 0.04), while tarsi did not significantly differ between North Kinangop and Kedong on day 1 or 7 (day 1, z = 0.60, *P* = 0.82; day 7, z = 1.49, *P* = 0.29).

#### Growth constants restricted to complete individual records

To further explore possible causes of the differences in growth constant K among locations, we restricted the data sets in each location to individual nestlings for which we had complete sets of repeated measurements (i.e., days 1, 4, 7, and 10; Kedong *n* = 10, North Kinangop *n* = 3, South Kinangop *n* = 10). With this approach we excluded nestlings that disappeared from the data set as a result of starvation, nest predation or flooding. Because we had observed nestlings in poor condition especially in South and North Kinangop, we hypothesized that selective disappearance of this subset of nestlings might have affected the difference in K**-**values among locations. Indeed, restricting the analyses to healthy nestlings that successfully grew and fledged, yielded increased K values in North Kinangop and South Kinangop, confirming our observations that nestlings died of poor condition in these locations, but these values remained lower than in Kedong (Table 3. Note: based on 95% confidence intervals, differences were not significant).

#### Immune function

**Fig. 2:**
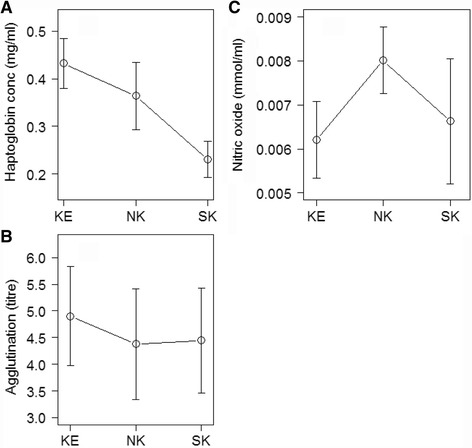
Haptoglobin concentration (mg/ml ± SE, **a**), agglutination (titre ± SE, **b**) and nitric oxide concentration (mmol/ml ± SE, **c**) of 10-day old nestlings of Red**-**capped Larks *Calandrella cinerea* in South Kinangop (SK, cool and wet), North Kinangop (NK, warm and wet) and Kedong (KE, warm and dry), three Kenyan localities differing in climatic conditions

Log haptoglobin (mg/ml) were highest in Kedong, intermediate in North Kinangop and lowest in South Kinangop (Fig. 2a), a non**-**significant location effect (F_2, 25_ = 2.84, *P* = 0.08). Log agglutination titre did not differ significantly among the three locations (Fig. 2b), (F_2, 26_ = 0.12, *P* = 0.88). Lysis titre was zero for 45 out of the 47 nestlings and lysis titre was one for two 10**-**day old nestlings, one individual each from South and North Kinangop. We therefore did not test for among**-**location differences in lysis. There was no location effect among the three locations in nitric oxide (mmol/ml) (Fig. 2c), (F_2, 23_ = 0.55, *P* = 0.59). Hatching order did not affect log haptoglobin (F_1, 14_ = 0.12, *P* = 0.73), log agglutination (F_1, 17_ = 0.01, *P* = 0.93) or nitric oxide (F_1, 10_ = 1.30, *P* = 0.28).

### Nestling growth but not immune function changes with population breeding intensity and rainfall in a year-round breeder in Kedong

**Fig. 3:**
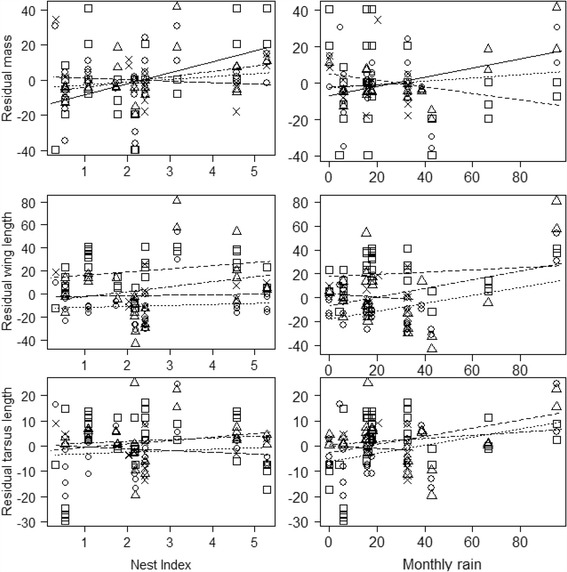
Mass, wing and tarsus lengths (residuals in % of the logistic growth curve) of four age-classes of nestlings of Red**-**capped Larks *Calandrella cinerea* as a function of nest index (number of nests/10 search hours) and monthly rainfall (mm) in Kedong (warm and dry), Kenya. Residual mass showed a significant relationship with nest index for nestlings at hatching (day 1) and with monthly rainfall for nestlings at day 7; both of these lines are represented by open squares and continuous line and open triangles and continuous line respectively. Legend for the non-significant relationship: open square and dotted line represent nestlings aged day 1, open circles and dotted line day 4, open triangles and dot-dashed line day 7, crosses and line with long dashes day 10

Analysing residual mass as a function of the nest index of the month of hatch, we found a significant interaction between nest index and age, and no significant effect of hatching order (Fig. 3, Table 5). Analyses per age showed that at hatching nestling body mass was higher when nest index was higher (day 1: F_1,21_ = 8.80, *P* = 0.01), but at days 4, 7, and 10 we observed no significant relation with nest index (all F < 2.3, all *P* > 0.15; Fig. 3). Analyses of residuals for mass with monthly rainfall also revealed a significant interaction between rainfall and age. When analysed per age, the analysis revealed a significant difference on day 7 (F_1,13_ = 5.78, *P* = 0.03, Table 5): with more rain, 7**-**day**-**old nestlings were heavier (Fig. 3). At other ages, mass did not correlate with rainfall (all F < 0.76, all *P* > 0.39; Fig. 3). At day 10, the range of rainfall values is limited, prohibiting robust evaluation at this age.

**Table 5:** Results of linear mixed**-**effect models examining the relationship of residuals for mass (in g) wing and tarsus lengths (in mm) in relation to A). nest index, age and chick hatching order and B). monthly rainfall (mm), age and chick hatching order, in Red**-**capped Lark *Calandrella cinerea* nestlings in Kedong

	d.f.	F	P
Mass			
Nest index*Age	3, 66	3.42	0.02
Nest index	1, 28	13.05	0.001
Age	3, 66	2.39	0.08
Hatching order	1, 65	0.14	0.71
Wing			
Nest index*Age	3, 64	0.87	0.46
Nest index	1, 28	1.30	0.26
Age	3, 67	35.71	<0.001
Hatching order	1, 26	0.58	0.45
Tarsus			
Nest index*Age	3, 65	1.81	0.15
Nest index	1, 28	0.30	0.59
Age	3, 68	2.25	0.09
Hatching order	1, 26	0.31	0.58
Body Mass			
Rainfall*Age	3,65	3.21	0.03
Rainfall	1,28	2.07	0.16
Age	3,65	1.41	0.25
Hatching order	1,26	0.14	0.71
Wing			
Rainfall*Age	3,64	2.01	0.12
Rainfall	1,28	0.41	0.53
Age	3,67	35.42	<0.001
Hatching order	1,26	0.55	0.47
Tarsus			
Rainfall*Age	3,65	2.07	0.11
Rainfall	1,28	1.42	0.24
Age	3,68	2.13	0.10
Hatching order	1,26	0.28	0.60

Wing and tarsus lengths were unrelated to nest index or rainfall in Kedong at any age (Fig. 3, Table 5). Wing and tarsus lengths were also unrelated to hatching order (Table 5).

#### Immune function

**Fig. 4:**
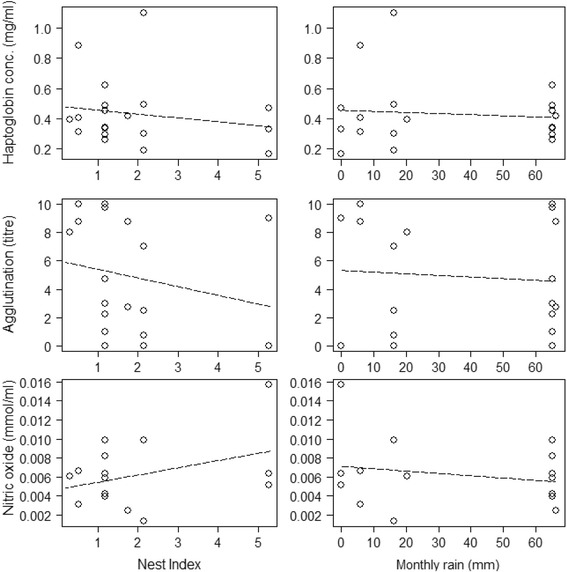
Haptoglobin (mg/ml), agglutination (titre) and nitric oxide (mmol/ml) of 10-day old nestlings of Red**-**capped Larks *Calandrella cinerea* as a function of nest index and monthly rainfall in Kedong (warm and dry), Kenya. *Dashed lines* means that the relationship is non-significant

The three immune indices were unrelated to nest index or rainfall in Kedong (Fig. 4): log haptoglobin (nest index F_1,10_ = 1.31, *P* = 0.28; rainfall F_1,10_ = 0.14, *P* = 0.71), log agglutination (nest index F_1,9_ = 1.37, *P* = 0.27, rainfall F_1,9_ = 0.11, *P* = 0.75) and nitric oxide (F_1,10_ = 2.85, *P* = 0.12, rainfall F_1,10_ = 0.79, *P* = 0.40). Similarly, hatching order was unrelated to log haptoglobin (F_1,6_ = 0.08, *P* = 0.78), log agglutination (F_1,6_ = 0.01, *P* = 0.94) or nitric oxide (F_1,3_ = 0.17, *P* = 0.70).

## Discussion

Red**-**capped Lark nestlings from three climatically**-**distinct, but geographically close tropical environments differed in growth in ways that suggest independently acting influences of female body condition and food availability for nestlings. Nestling body mass and size at hatching, which presumably reflect the resources that females allocated to their eggs [14, 59–61], were lowest in the most arid location, Kedong. However, contrary to our predictions, nestlings in this location grew faster than in the other two cooler and wetter locations of South and North Kinangop. Consistent with these among**-**location findings, the differences in growth of nestlings at different times of the year within Kedong, although partially in contrast with our predictions, also pointed to independent effects of female body condition and food availability for nestling growth. At times of the year when more individuals in the Kedong population bred, suggesting conditions for breeding for females were favorable, nestlings had indeed higher body mass at hatching, but they did not grow better in the days thereafter. Where we previously had found that rainfall did not affect the timing of breeding [30], we now discovered that rainfall also had no effect on nestling mass at hatching, but that more rain did coincide with faster growth post**-**hatch. Finally, and unexpectedly, neither the among**-**location comparison, nor the within**-**location analysis in Kedong revealed any significant differences in nestling immune function. The results on the variation in nestling growth rate demonstrate the strong roles of female body condition and that of food availability in defining the pace-of-life and variation in life-history strategies within-tropical environments.

Slow growth rates in the tropics have been attributed to the poor food quality and low food availability [62], and our within**-**tropics study also implicates a role of food in explaining differences in growth. Whereas increasing aridity has been associated with slower growth [10], growth of Red**-**capped Larks in our study was actually fastest in the most arid location (Kedong). We propose that the local ecology of the three locations in the current study may not represent a typical aridity gradient with a decrease in primary productivity, and an associated decrease in invertebrates. In a related study, [30] – unexpectedly – found that flying invertebrate biomasses were highest in the most arid location, Kedong, and lowest in the cooler and wetter locations, South and North Kinangop. The large amounts of precipitation in South Kinangop, and to a lesser extent in North Kinangop, led to frequent flooding, which we hypothesized negatively impacted the food quality and quantity for nestling larks, with negative consequences for growth [3, 63, 64]. When we fitted growth curves based on data for nestlings that we monitored from hatching to fledging, instead of including all nestlings irrespective of their fate, the growth constants K, increased in South and North Kinangop, but not in Kedong. We interpret this result as additional evidence of food limitation for nestlings in South and North Kinangop, but not in Kedong. Inclusion of nestlings that may have starved to death before getting to fledging age in South and North Kinangop pulled down the K value for these locations. The increased growth constant when restricting analyses to nestlings that fledged, adds to the likelihoodthat food is a more important factor in determining nestling growth rate (and immune function) than thermoregulation in this study system.

The within**-**Kedong findings that nestlings hatched at a higher mass when more larks were breeding, but grew better in times with more rain (that do not coincide with higher numbers of breeding females) raise the question why females did not preferentially breed at times that were best for nestling growth. Numerous studies, mostly of nest**-**box breeding birds in temperate zones, have shown that females time breeding such that nestlings benefit optimally from the food peak that is common in temperate zone springs [64–67]. Our results suggest that the timing of breeding by Red**-**capped Larks is affected by other factors than food, for example nest predation, protein reserves of individual females or social factors [30]. However, the success of these birds’ breeding attempts is at least partly determined by environmental factors, such as rain, that typically correlate with food availability. A lesser role for food may also be concluded from the small and typically constant clutch size of two eggs that fits with a bet**-**hedging strategy in a high**-**risk environment [68].

A comparison among growth constants (K) of larks from other regions in the world [10] indicates that our within**-**tropical variation in growth of Red**-**capped Larks spans a range similar to, but lower than the range found among a variety of lark species from different environments. K varied from 0.34 to 0.54 in Red**-**capped Larks within the tropics (Table 3), and from 0.41 to 0.62 along a gradient from desert to temperate environments [10]. In general, neotropical passerines have been shown to grow 23% slower than temperate birds [3], but within**-**tropics variation has not received much attention. In contrast with many tropical species [3, 33], but in line with other lark species [10], Red**-**capped Larks did not show hatching asynchrony: hatching order did not affect nestling size, growth or immune function.

The three measured immune indices, haptoglobin, agglutination, and nitric oxide, showed more variation within than among locations. Furthermore, while varying substantially among individual nestlings, the indices did not significantly covary with nest index or rain within Kedong. The reliance on measurements at a single time point (i.e., day 10) can complicate comparisons among locations and through time, since the immune systems of nestlings, including components measured in the current study, may develop at different rates across individuals or populations e.g., [69]. Immunity of nestlings may partly reflect the immunological status of their parents [38, 39, 70].The fact that only two nestlings had lysis (45 out of 47 nestlings had a value of zero) also supports the idea that the nestling immune system is not yet fully developed, a finding consistent with other studies [39, 71]. When comparing haptoglobin in 10**-**day old tropical Red**-**capped Lark nestlings (Fig. 2) with 8**-**day old temperate Skylarks (0.28 mg/ml, [71], haptoglobin values for the latter are closest to the tropical South Kinangop population, which were about twice as low as Kedong and North Kinangop (although the difference was statistically insignificant). Of the three tropical locations, South Kinangop’s relatively cool and wet conditions resemble most closely a temperate environment. Agglutination titres of the Skylark nestlings (2.45) are lower than in all three Red**-**capped Lark populations (Fig. 2). Combined with the observation that agglutination of adults is higher in Skylarks than in Red**-**capped Larks [21], this finding suggests among**-**species differences in the ontogeny of their immune systems.

## Conclusion

In conclusion, we found that nestlings of Red-capped Larks differed in size at hatching and growth rate among three climatically-distinct tropical locations and with year-round variation within location in Kedong. We propose that female quality and resource availability played independent roles in determining these findings. Whereas females in the resource**-**scarce cool and wet locations [30] presumably allocated more resources to their eggs, giving rise to nestlings with larger body mass and size at hatching, parent birds in the more arid but resource**-**abundant Kedong [30] committed more resources to feeding nestlings post-hatch, leading to higher growth rates. In addition, nestlings hatched during times of the year when more individuals were breeding in Kedong, presumably when conditions for breeding for females were favorable, had higher body mass at hatching. Parents in the different locations and at different times of the year in the same location, apply different life-history strategies (adjustment of female body condition and utilization of food availability) leading to differences in the pace-of-life. Innate immunity did not vary among locations and within Kedong it did not co**-**vary with nesting intensity or rain. Because we measured the immune system at one specific time point (day 10) only, it would be interesting to study the entire development trajectory of the nestling immune system and compare potential differences among and within populations, in relation to environmental conditions.

## Appendix

**Table 6:** Mean (±SE), the pairwise locational differences, the mean of the means of males and females and sample sizes per location for adult Red**-**capped Lark *Calandrella cinerea* that we studied from January 2011 to March 2014

Variable	Location	Mean ± SE, with pairwise locational differences (*P* < 0.05)		Mean of males and females	Sample sizes per sex	
		Females	Males		Females	Males
Mass (g)	South Kinangop	25.9 ± 0.31^a^	25.8 ± 0.54^ab^	25.9	34	17
	North Kinangop	25.5 ± 0.25^a^	25.7 ± 0.39^a^	25.6	39	27
	Kedong	24.1 ± 0.18^b^	24.4 ± 0.30^b^	24.3	100	53
Wing length (mm)	South Kinangop	90.2 ± 0.46^a^	94.8 ± 0.74^a^	92.5	36	17
	North Kinangop	89.9 ± 0.46^ab^	94.1 ± 0.60^ab^	92.0	39	27
	Kedong	88.9 ± 0.20^b^	92.8 ± 0.37^b^	90.9	100	55
Tarsus (mm)	South Kinangop	25.3 ± 0.20^ab^	25.9 ± 0.26^a^	25.6	36	19
	North Kinangop	25.4 ± 0.20^a^	25.4 ± 0.23^ab^	25.4	39	26
	Kedong	24.9 ± 0.10^b^	25.2 ± 0.13^b^	25.1	100	55

Different letters indicate significant within sex differences among locations (not between sexes within location) in the post**-**hoc tests
